# Fasting and meal-related zonulin serum levels in a large cohort of obese children and adolescents

**DOI:** 10.3389/fendo.2024.1329363

**Published:** 2024-02-08

**Authors:** Giorgia Pepe, Domenico Corica, Monica Currò, Tommaso Aversa, Angela Alibrandi, Riccardo Ientile, Daniela Caccamo, Malgorzata Wasniewska

**Affiliations:** ^1^ Department of Human Pathology of Adulthood and Childhood, University of Messina, Messina, Italy; ^2^ Department of Biomedical and Dental Sciences and Morpho-Functional Imaging, University of Messina, Messina, Italy; ^3^ Department of Economics, University of Messina, Messina, Italy

**Keywords:** zonulin, childhood obesity, intestinal permeability, insulin resistance, glucose homeostasis

## Abstract

**Introduction:**

Zonulin recently emerged as a valuable biological marker to assess the integrity of the intestinal mucosal barrier. Nevertheless, data about zonulin in pediatric age are extremely scarce. Aim of this study was to investigate the relationship between serum zonulin levels, both fasting and postprandial, with body mass index (BMI) and biochemical markers of insulin resistance (IR), insulin sensitivity, b-cell function and cardio-metabolic risk in obese non-diabetic youths.

**Methods:**

One hundred and four children and adolescents with obesity (BMI ≥ 2.0 SDS) were enrolled (mean age 11.43 ± 2.66). All the patients underwent clinical and biochemical assessment, including oral glucose tolerance test (OGTT) and liver ultrasonography. Zonulin serum levels were measured at fasting state, at 60-minute and 120-minute OGTT timepoint.

**Results:**

Impaired fasting glycaemia and impaired glucose tolerance were documented in 27.9% and 11.5% of patients, respectively. IR was documented in 69.2% of cases. Liver steatosis was diagnosed in 39.4%. Zonulin serum levels significantly increased from baseline to 60-minute and 120-minute OGTT timepoint (p positive correlation between BMI SDS and serum zonulin levels at 120-minute OGTT timepoint (p highlighted a positive association of zonulin fasting levels with IR and glutamicoxalacetic transaminase levels (GOT, p zonulin levels were demonstrated for age, sex, pubertal status, glucose, lipid profile and the other obesity-related parameters.

**Discussion:**

Our results show, for the first time in a pediatric cohort, the meal-related pattern of secretion of serum zonulin, which tends to significantly increase during and at 2-hours postprandial assessment. Even if the underlying mechanisms associating intestinal permeability and obesity have not been fully elucidated yet, our data confirm a close relationship between zonulin concentration and obesity in pediatric population. IR seems to significantly influence zonulin serum levels, thus a central role of IR in this pathway is conceivable.

## Introduction

Due to its recently documented role in reversible intercellular tight junction disassembly, Zonulin has emerged as a valuable and promising biological marker to assess the integrity of the intestinal mucosal barrier. Human zonulin (47 KDa protein), which was discovered as an analogue of the cholera comma toxin (ZOT, zonula occludens toxin), is secreted mainly by the liver and the enterocytes, and can be isolated from multi-protein membrane complexes (claudin-occludin-guanylate kinase-like proteins ZO-1, ZO-2, and ZO-3) on the apical surface of intestinal epithelium. It represents the only measurable blood protein known to regulate the permeability of intestinal tight junctions, enabling the paracellular transport in the gut’s mucosa (1–4).

Dysregulation of the zonulin pathway leads to increased intestinal permeability, which may influence tolerance and immunity. High serum levels of zonulin and the subsequent condition of “gut leakiness” have been associated with both intestinal and extraintestinal disorders, including autoimmune diseases (such as celiac disease and type 1 diabetes mellitus), cancers and diseases of the nervous system. Interestingly, genes related to these three classes of diseases mapped on chromosome 16, the same where zonulin gene is located (2, 5–8). Moreover, recent evidence highlighted a potential role of zonulin in the pathophysiology of obesity. Elevated circulating zonulin concentrations seem to positively correlate with body mass index (BMI), glucose levels, dyslipidemia, systolic blood pressure (SBP), and insulin resistance (9–12). Some authors reported significantly higher levels of zonulin in prediabetic and diabetic patients (13) and in pregnant women with gestational diabetes mellitus (14–16). Even if the underlying mechanisms associating serum zonulin level with obesity are still unknown, experimental studies have shown a close relationship between intestinal permeability and obesity (17–19). In this regard, it has been hypothesized that a modification in the gut microbiota – secondary to a continuous uptake of high-fat diet - may promote chronic inflammation of the gut, and subsequently at a systemic level (20–22). This chronic low-grade systemic inflammation, which characterizes obesity, upregulates zonulin expression and may be an important contributor to intestinal barrier dysfunction (3, 23). Elevated serum zonulin levels have been also associated with increased liver enzymes (24) and non-alcoholic fatty liver disease (NAFLD), shedding new light on the so called “gut-liver-axis” (25–30).

To the best of our knowledge only few studies have investigated the link between and obesity in pediatric age (24, 25, 31–33). No data are currently available about the meal-related pattern of secretion of zonulin in pediatric age. Aim of the study was to investigate the relationship between fasting and postprandial serum zonulin concentration, BMI, and obesity-related biochemical markers in non-diabetic children and adolescents with obesity.

## Materials and methods

This single-center and cross-sectional study was carried out from November 2020 to October 2023. Children and adolescents were enrolled according to the following inclusion criteria: chronological age between 5 and 16 years; BMI ≥ +2.0 standard deviation score (SDS), according to the definition of obesity proposed by the World Health Organization (WHO) for children from the age of 5 years (34); caucasian ethnicity; born full-term and adequate for gestational age (AGA). Criteria of exclusion from the study were: pre-term or post-term birth, diabetes, genetic or endocrine causes of obesity, chronic diseases or chronic pharmacological therapies, smoking.

### Clinical and biochemical assessment

At recruitment, patients underwent a comprehensive medical history, specifically focused on family or personal history of cardio-metabolic risk factors. Auxological assessment was based on height evaluation and BMI calculation. Physical examination was performed by a dedicated team of pediatric endocrinologists, including measurement of height, weight, BMI, waist circumference (WC), WC-to-height ratio (WHtR), systolic and diastolic blood pressure. Standing height was measured with a Harpenden stadiometer (Holtain Ltd, Crymych, Dyfed, UK). BMI was calculated using the equation: body weight(kg)/height(m)^2^. To allow the comparison between different ages and genders, height and BMI were expressed as Standard deviation scores (SDS). Waist circumference (WC) was measured to the nearest 0.5 cm while the subjects were standing, as previously described (35). SBP and diastolic blood pressure (DBP) were recorded at rest on the right arm, using a manual sphygmomanometer; for analysis, the average of three blood pressure values was calculated. Pubertal stage was assessed according to the Tanner classification (36).

All the patients underwent fasting biochemical assessment, including blood sampling for lipid profile, thyroid, kidney and liver function blood tests and oral glucose tolerance test (OGTT), performed at least 8 hours after the last meal, as previously described (37). OGTT was performed according to the American Diabetes Association (ADA) guidelines (38), with sampling at 0, + 30, + 60, +90, +120 minutes for measurements of glucose and insulin. Impaired fasting glycaemia (IFG) was defined as fasting plasma glucose between 100 and 125 mg/dL. Impaired glucose tolerance (IGT) was defined as plasma glucose values between 140 and 199 mg/dL two hours after a standardized glucose load (OGTT) (38).

Homeostasis model assessment of insulin resistance (HOMA-IR), β-cell function (HOMA-B), Matsuda-index, Insulinogenic-index were calculated. Areas Under the Curves for glucose (AUCg) and insulin (AUCi) and their ratio were also evaluated. HOMA-IR was calculated according to the equation: fasting insulin (μU/ml) × fasting glucose (mg/dl)/405 (39). The aterogenic index of plasma (AIP) was calculated according to the equation: [LOG (triglycerides/HDL cholesterol)] (40). The following cut-offs were used to define the main indices of cardiometabolic risk as abnormal: HOMA-IR>2.5 in prepubertal children and HOMA-IR>4 in pubertal patients (39), AIP >0.11 (40), and TG/HDL ratio >1.25; HbA1c was defined as normal below 6% (42 mmol/l or 756 mg/dl) (41, 42).

Blood samples for the serum zonulin assay were taken at fasting state, at 60-minute and 120-minute OGTT timepoint. Samples for the serum zonulin assay were collected and stored at −80°C. Concentrations of zonulin were measured by using competitive enzyme-linked immunosorbent assay (ELISA) kits (K5600, Immundiagnostik AG, Bensheim, Germany) according to the manufacturer’s instructions. The absorbance values for the ELISA assays were determined with an Inflinite 2000 Pro multimode plate reader (Tecan, Vienna, VA, USA) at 450nm.

Liver ultrasonography (US) was performed in all the recruited subjects. Liver steatosis was diagnosed by conventional liver US according to at least two of the following criteria: 1) diffused and increased echogenicity of the liver compared with kidney or spleen; 2) US beam attenuation; 3) scarce visualization of intrahepatic structures (43).

### Statistical analysis

Numerical data were expressed as mean, SDS, median and interquartile range (Q1-Q3); categorical variables were expressed as absolute frequencies and percentage. Non-parametric approach was used since the numerical variables were not normally distributed, such as verified by the Kolmogorov-Smirnov test. The Mann Whitney test was applied with reference to numerical parameters in order to identify possible significant differences between the different groups of subjects divided according to the following parameter: sex, pubertal stage, fasting glucose, glucose tolerance, HbA1c levels, IR, liver steatosis. The Chi Square test was applied to compare the same above-mentioned groups with regards to categorical variables. The Spearman correlation test was applied to assess the correlation between zonulin level at 0’, 60’ and 120’ minutes with numerical variables.

Friedman test was applied to evaluate the trend of zonulin serum levels in the three analyzed OGTT timepoints (0’, 60’ and 120’ minutes). In addition, all two-by-two comparison were performed using Dunn test; for this analysis the Bonferroni’s correction was applied by dividing the significance level (0.050) into the total number of the possible two-by-two comparisons; as a result, the adjusted significance level was 0.017. The Mann-Kendall statistical test for trend was used to assess whether our set of data values was increasing over time or decreasing over time, and whether the trend in either direction was statistically significant.

A multiple stepwise regression model was estimated in order to identify significant predictors of zonulin serum level at fasting state; in particular, we tested the influence of the following covariates: sex, age, BMI, waist circumference, glutamic oxaloacetic transaminase (GOT), glutamaic pyruvic transaminase (GPT), total cholesterol, triglycerides, liver steatosis, HbA1c, C-reactive protein (CRP), insulin resistance, impaired fasting glycaemia (IFG), impaired glucose tolerance (IGT). Results were expressed as regression coefficient (b), 95% confidence interval (C.I.) and p-value. A boxplot and a scatterplot were built to allow the visualization of the results of the regression analysis.

Statistical analyses were performed using IBM SPSS for Windows, Version 22 (Armonk, NY, IBM Corp.). A *p*-value lower than 0.05 was considered statistically significant.

## Results

### Main clinical and biochemical data

One hundred and four children and adolescents (48 males and 56 females) with obesity were enrolled. At recruitment, the mean age was 11.43 ± 2.66 years; 68.3% of them were pubertal and 31.7% prepubertal. The main clinical and biochemical characteristics of the study population are detailed in **Table 1**. Overall, the mean BMI of the study population was 29.29 ± 4.3 SDS. Impaired fasting glycaemia (IFG) was documented in 27.9% (29 patients). After OGTT load, 11.5% of patients (12 subjects) were diagnosed with impaired glucose tolerance (IGT). IR was documented in 69.2% patients (72 subjects). Liver steatosis was diagnosed in 39.4% of patients (41 subjects). The degree of the steatosis was described ultrasonographycally as mild in 27.9%, moderate in 8.7% and severe in 2.9% of cases. An increase in liver volume was reported in 23.1% of patients. A comparison analysis was performed in the entire cohort for sex and for pubertal status (pubertal/prepubertal) without evidence of significant difference in zonulin levels, both at fasting state and postprandial. Likewise, no differences were documented in zonulin levels when comparing subjects with IFG vs normal fasting glycaemia, impaired vs normal glucose tolerance, high vs normal HbA1c and presence/absence of liver steatosis. When obese patients were categorized in IR and non-IR subjects according to HOMA-IR, no significant difference in zonulin fasting and postprandial levels were recorded (**Table 2**). Overall, we found a higher rate of IFG in pubertal patients (p= 0.048). Liver steatosis was significantly more frequent in boys than in girls (p=0.037) and in IR subjects than in non-IR ones (p=0.054).

**Table 1:** Clinical and biochemical features of the study population.

	Mean	SDS
**Age (years)**	11.43	2.66
**Height (SDS)**	0.52	1.09
**Weight (SDS)**	2.20	0.55
**BMI**	29.29	4.30
**BMI (SDS)**	3.06	0.71
**WC (cm)**	86.30	15.67
**HC (cm)**	96.01	18.14
**WC/HC**	0.90	0.06
**WHtR**	0.59	0.05
**SBP (mmHg)**	114.34	8.79
**DBP (mmHg)**	69.89	8.69
**GOT (U/L)**	21.02	8.41
**GPT (U/L)**	23.66	20.62
**GGT (U/L)**	16.05	16.55
**Total cholesterol (mg/dl)**	169.92	25.89
**LDL-cholesterol (mg/dl)**	89.71	27.19
**HDL-cholesterol (mg/dl)**	52.69	15.67
**Triglycerides (mg/dl)**	84.58	36.76
**Triglycerides/HDL-ratio**	1.80	1.15
**Total cholesterol/HDL-ratio**	3.47	1.19
**Uric acid (mg/dl)**	4.93	1.21
**CRP (mg/dl)**	0.99	6.21
**HbA1c (%)**	5.33	0.44
**Fasting glucose (mg/dl)**	95.83	8.12
**1h-postprandial glucose (mg/dl)**	129.58	21.86
**2h-postprandial glucose (mg/dl)**	114.92	16.85
**Fasting insulin (mUI/ml)**	21.16	12,06
**1h-postprandial insulin (mUI/ml)**	131.70	108.25
**2h-postprandial insulin (mUI/ml)**	121.41	89.23
**HOMA-IR**	5.05	3.10
**HOMA-B**	237.82	131.48
**IGI**	2.89	2.77
**Matsuda-index**	2.50	1.28
**AUCg**	260.18	152.05
**AUCi**	245.29	217.30
**AUCi/AUCg ratio**	0.93	0.61
**FT4 (pmol/L)**	15.29	2.52
**TSH (uUI/ml)**	2.57	2.60
**Fasting Zonulin (pg/ml)**	19.26	12.53
**1h-postprandial Zonulin(pg/ml)**	21.04	15.38
**2h-postprandial Zonulin(pg/ml)**	21.75	16.027

Numerical data are expressed by mean ± SDS.

BMI, Body mass index; SDS, standard deviation score; WC, waist circumference; HC, hip circumference; WHtR, WC-to-height ratio; SBP, systolic blood pressure; DBP, diastolic blood pressure; GOT, glutamic-oxaloacetic transaminase; GPT, Glutamate Pyruvate Transaminase; GGT, gamma-glutamyl transpeptidase; CRP, C-reactive protein; HbA1c, glycated haemoglobin; HOMA-IR, homeostasis model assessment of insulin resistance; HOMA-B, homeostasis model assessment for β-cell function; IGI, insulinogenic index; AUC_g_, Area under the curve for glucose; and AUC_i_ insulin. TSH, thyroid stimulating hormone; FT4, free thyroxine.

**Table 2:** Comparison analysis between insulin resistant (IR) and non-insulin resistant subjects.

	IR subjects (n=72)	Non-IR subjects (n=32)	p-value
**Age (years)**	11.44 ± 2.54	11.39 ± 2.95	
**Sex (M/F)**	32/40	16/16	0.600
**Height (SDS)**	0.65 ± 1.04	0.25 ± 1.18	**0.035**
**Weight (SDS)**	2.28 ± 0.56	2.05 ± 0.52	**0.025**
**BMI**	29.72 ± 4.38	28.33 ± 4.03	0.220
**BMI (SDS)**	2.24 ± 0.49	2.11 ± 0.45	0.214
**WC (cm)**	87.70 ± 14.41	83.20 ± 18.04	0.173
**HC (cm)**	97.84 ± 16.83	91.97 ± 20.48	0.076
**WC/HC**	0.90 ± 0.06	0.92 ± 0.08	0.144
**WHtR**	0.59 ± 0.05	0.58 ± 0.05	0.648
**SBP (mmHg)**	114.98 ± 9.16	112.96 ± 7.94	0.253
**DBP (mmHg)**	70.44 ± 9.28	68.74 ± 7.31	0.283
**GOT (U/L)**	21.31 ± 9.69	20.40 ± 4.50	0.504
**GPT (U/L)**	25.82 ± 23.41	18.87 ± 11.33	0.080
**GGT (U/L)**	17.82 ± 19.38	11.82 ± 3.02	**0.015**
**Total cholesterol (mg/dl)**	169.73 ± 27.56	170.34 ± 22.14	0.542
**LDL-cholesterol (mg/dl)**	92.80 ± 27.55	83.16 ± 25.60	0.137
**HDL-cholesterol (mg/dl)**	50.60 ± 13.85	57.34 ± 18.52	**0.032**
**Triglycerides (mg/dl)**	88.42 ± 39.40	76.06 ± 28.87	0.163
**Triglycerides/HDL-ratio**	1.90 ± 1.11	1.59 ± 1,24	0.055
**Total cholesterol/HDL-ratio**	3.53 ± 0.92	3.35± 1,66	0.101
**Uric acid (mg/dl)**	4.95 ± 1.19	4.87 ± 1.30	0.795
**CRP (mg/dl)**	1.36 ± 7.41	0.14± 0.11	**0.003**
**HbA1c (%)**	5.37 ± 0.33	5.25 ± 0.62	**0.025**
**Fasting glucose (mg/dl)**	97.04 ± 7.70	93.12 ± 8.53	**0.012**
**1h-postprandial glucose (mg/dl)**	130.79 ± 20.21	126.90 ± 25.28	0.410
**2h-postprandial glucose (mg/dl)**	117.61 ± 16.62	108.97 ± 16,06	**0.012**
**Fasting insulin (mUI/ml)**	25.65 ± 11.76	11.08 ± 3.97	0.000
**1h-postprandial insulin (mUI/ml)**	146.19 ± 123.01	99.56 ± 53.30	**0.012**
**2h-postprandial insulin (mUI/ml)**	141.05 ± 98.82	79.06 ± 39.48	**0.000**
**HOMA-IR**	6.19 ± 3.07	2.52 ± 0.89	**0.000**
**HOMA-B**	278.53 ± 130.12	146.24 ± 78.48	**0.000**
**IGI**	3.19 ± 3.21	2.2523 ± 1.159	0.097
**Matsuda-index**	1.98 ± 0.86	3.68 ± 1.30	**0.000**
**AUCg**	269.55 ± 181.16	239.13 ± 31.62	**0.047**
**AUCi**	282.08 ± 249.55	162.54 ± 63.63	**0.000**
**AUCi/AUCg ratio**	1.04 ± 0.69	0.67 ± 0.24	**0.000**
**FT4 (pmol/L)**	15.10 ± 2.62	15.73 ± 2.21	0.310
**TSH (uUI/ml)**	2.78 ± 3.09	2.15 ± 0.79	0.770
**Fasting Zonulin (pg/ml)**	20.33 ± 13.93	16.88 ± 8.29	0.526
**1h-postprandial Zonulin(pg/ml)**	22.86 ± 17.45	17.48 ± 9.43	0.185
**2h-postprandial Zonulin(pg/ml)**	23.68 ± 18.39	17.92 ± 8.81	0.259
**Liver steatosis (n)**	32/64	9/30	**0.054**

Numerical data are expressed by mean ± SDS.

BMI, Body mass index; SDS, standard deviation score; WC, waist circumference; HC, hip circumference; WHtR, WC-to-height ratio; SBP, systolic blood pressure; DBP, diastolic blood pressure; GOT, glutamic-oxaloacetic transaminase; GPT, Glutamate Pyruvate Transaminase; GGT, gamma-glutamyl transpeptidase; CRP, C-reactive protein; HbA1c, glycated haemoglobin; HOMA-IR, homeostasis model assessment of insulin resistance; HOMA-B, homeostasis model assessment for β-cell function; IGG, insulinogenic index; AUC_g_, Area under the curve for glucose and AUC_i_ insulin. TSH, thyroid stimulating hormone; FT4, free thyroxine.

### The meal-related pattern of serum zonulin and its relationship with obesity-related biomarkers

**Figure 1:**
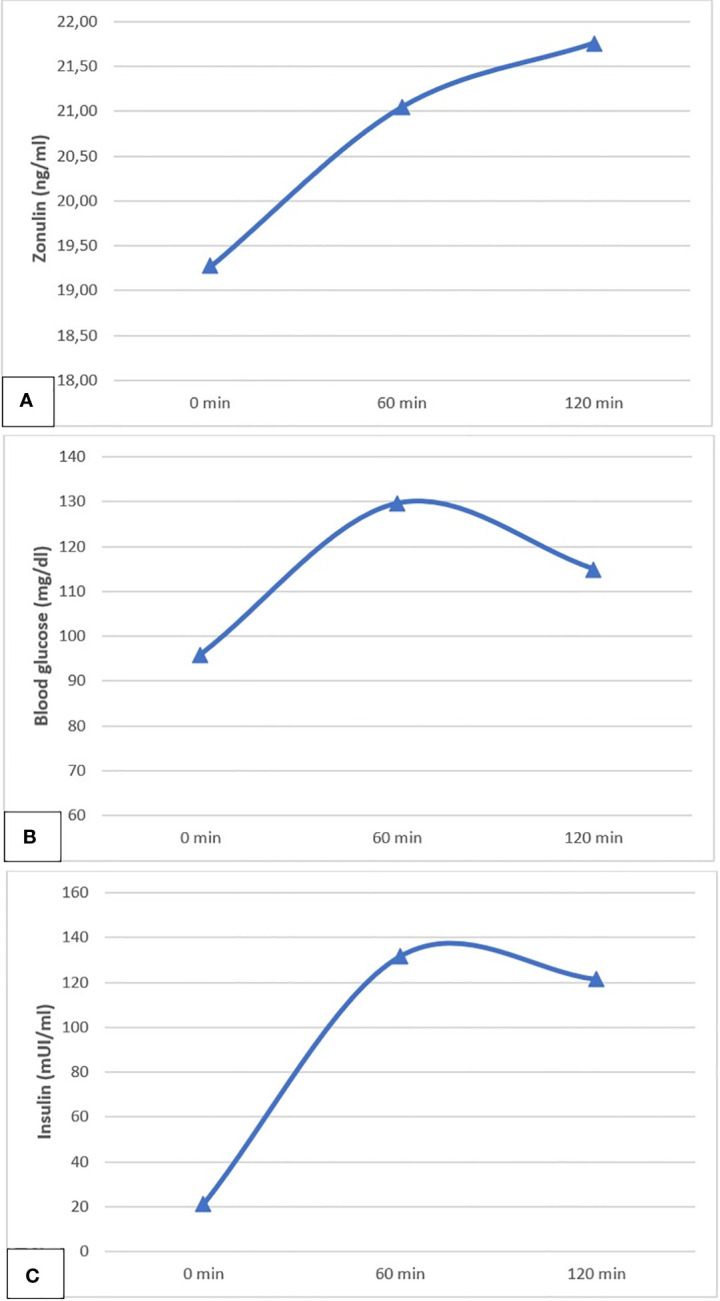
The meal related pattern of secretion of zonulin **(A)**, blood glucose **(B)** and insulin **(C)**, evaluated from baseline to 60 minutes and 120 minutes after oral glucose tolerance test (OGTT).

Zonulin serum levels significantly increased from fasting state to 60-minute and 120-minute OGTT timepoint (p<0.001) in the entire study population (**Figure 1A**). Such statistically significant increasing trend of zonulin secretion over time was confirmed by the Mann-Kendall test for trend (p=0.000). Conversely, blood glucose and insulin curves did not show a statistically significant trend (p=0.184 and p=0.168 respectively) in the entire cohort. Indeed - as expected - blood glucose and insulin levels reached a peak after 60 minutes after OGTT load, followed by a postprandial decrease from 60 to 120 minutes, showed in **Figures 1B, C**.

**Figure 2:**
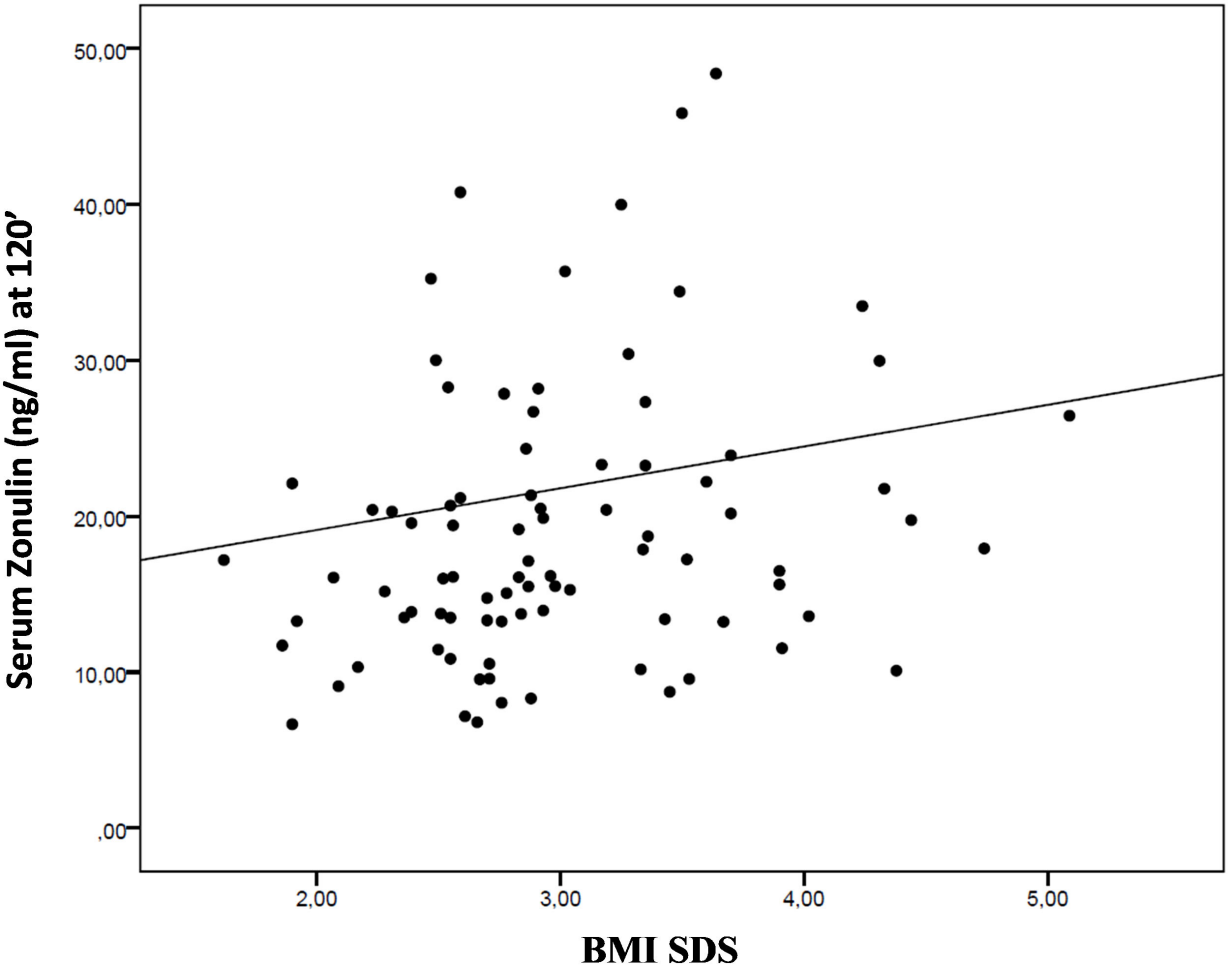
The scatter plot shows the interdependence between body mass index (BMI) SDS and serum zonulin levels measured at 120 minutes after oral glucose tolerance test (OGTT).

A positive correlation was demonstrated between BMI SDS and serum zonulin levels measured at 120-minute after OGTT load (r=+0.208, p=0.046), as showed in **Figure 2**.

**Figure 3:**
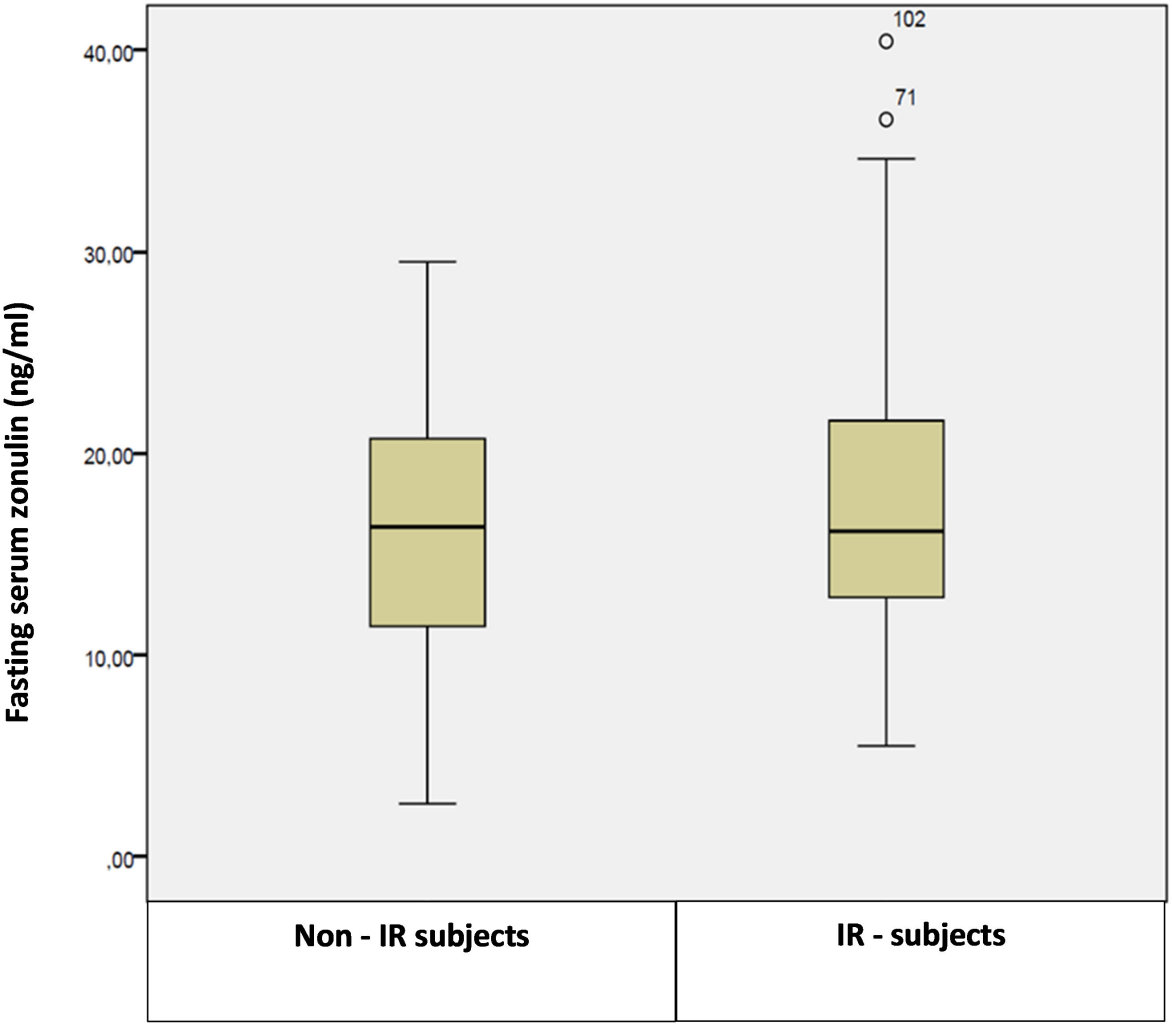
Box plot of fasting serum zonulin levels in insulin-resistant (IR) and non-insulin resistant patients.

**Figure 4:**
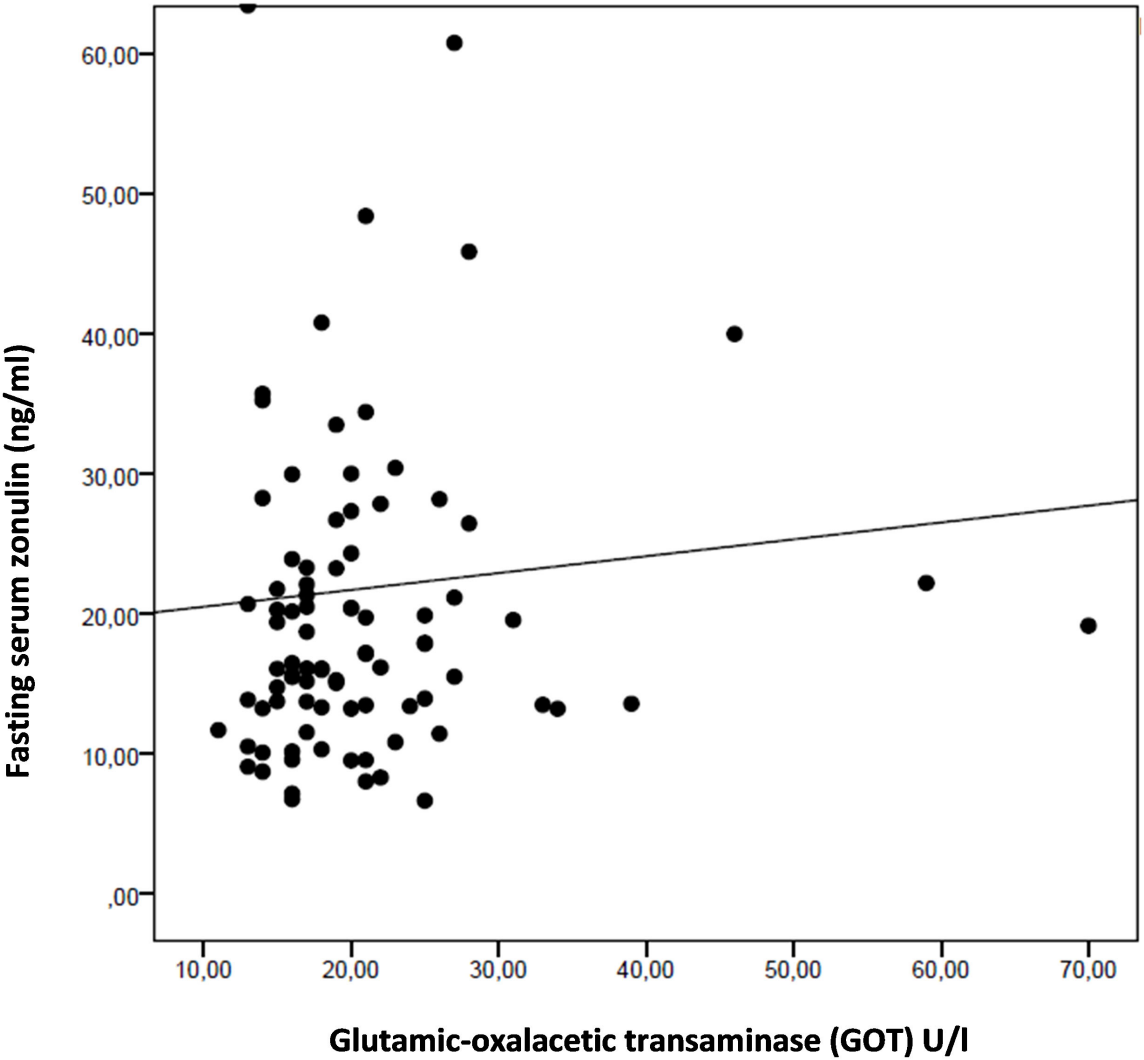
The scatterplot highlights a positive association between fasting serum zonulin levels and glutamic-oxalacetic transaminase (GOT) in the entire study population.

Multiple linear regression model highlighted a significant positive association of zonulin fasting levels respectively with IR (p = 0.039) and glutamic-oxalacetic transaminase (GOT) levels (p = 0.038), as showed in **Figures 3** and **4**. No significant differences in serum zonulin levels were demonstrated for age, sex, pubertal status, glucose, lipid profile and the other studied clinical and biochemical variables.

## Discussion

To the best of our knowledge, this is the first study to investigate the meal-related pattern of secretion of serum zonulin in a pediatric large cohort of non-diabetic children and adolescents with obesity.

According to the available literature reports, serum zonulin could be regarded as a novel biomarker of intestinal permeability and is suggested to play a role in the development of metabolic syndrome found in obese patients. Recently, higher serum zonulin levels were reported in obese subjects in comparison with healthy controls. These findings are well-demonstrated in adulthood (9–12). Conversely, only few studies investigated the relationship between zonulin secretion and obesity-related biomarkers in pediatric age (24, 25, 31, 33).

The results of the present study confirm a close relationship between serum zonulin concentration and the degree of obesity in pediatric age. Indeed, we documented a positive correlation between zonulin levels and BMI SDS in the entire study population. This finding is consistent with previous pediatric reports (24, 32, 33). In addition, we found a positive association between zonulin fasting levels and IR. Studies investigating the relationship between zonulin concentration and IR have yielded different results (9–11). Moreno-Navarrete et al. explored for the first time this relationship in adulthood, describing increased circulating zonulin levels in patients with glucose intolerance and obesity-associated IR versus controls, and enhancing the role of subclinical inflammatory cytokine IL-6 in this pathway (10). Likewise, Zhang et al. reported higher serum zonulin levels in adults with diabetes mellitus type 2 (T2DM) than in impaired or normal glucose tolerant subjects (11). In pediatric population, Kim et al. confirmed a positive association between zonulin concentration and BMI, fasting insulin, and HOMA-IR (24). Conversely, other authors did not find that IR was associated with serum zonulin levels (11, 31, 33). Moreover, in our cohort we found a positive association between fasting zonulin and GOT levels. The association between zonulin and liver enzymes in childhood obesity was reported also by Kim et al., who found a positive association with GPT levels (24). In addition, other studies reported that circulating zonulin is increased in children and adolescents with non-alcoholic fatty liver disease (NAFLD) and seems to correlate with the severity of liver steatosis (25), which we did not find in our study. Likewise, zonulin seems to correlate also with the risk of progression from liver steatosis to fibrosis. In this regard, Parkhomenko et al. reported a significant positive correlation between serum zonulin and PNFI (pediatric non-alcoholic fatty liver disease fibrosis index) in adolescents with obesity. Recently, an increasing number of studies demonstrated a close connection between the gut microbiota and the liver, shedding new insights on the gut-liver axis (25–28, 30). In particular, the results of intestinal permeability studies confirmed that impairment of intestinal wall integrity may play a pivotal role in the development and progression not only of obesity itself, but also of obesity-related metabolic disorders, such as liver steatosis and NAFLD (25, 28, 30, 32). However, impairment of intestinal permeability is not easily assessable. Nowadays, serum zonulin assay seems to become a reliable and easy way to reflect dysfunctional intestinal barrier, since zonulin is acknowledged as the only measurable blood protein to reversibly regulate intestinal permeability through modulation of intercellular tight junctions (4). It has been hypothesized that intestinal dysbiosis, secondary to high-fat and low-fibre diet, is a trigger for the increased synthesis of zonulin by the intestinal epithelium. Zonulin secretion leads to an increase in gut permeability secondary to the disassembly of the zonula occludens-1 from the tight junctional complex (44). Such an increased intestinal permeability may allow bacterial translocation, as well as promoting chronic inflammation of the gut, followed by systemic inflammation. This chronic low-grade systemic inflammation, which characterizes obesity, is an important contributor to intestinal barrier dysfunction and might in turn upregulate zonulin expression (17, 21, 22, 45).

Our results highlighted, for the first time in a pediatric cohort, the meal-related pattern of secretion of serum zonulin, which tends to significantly increase during and at 2-hours postprandial assessment. The increasing trend exhibited by zonulin did not reflect the dynamics of the glycemic and insulinemic curves, which instead are characterized by a 60-minute postprandial peak followed by decreasing trend documented at the 120-minute assessment. These data highlight the effect of hyperglycemic stress induced by oral glucose tolerance testing (OGTT) on zonulin secretion in our cohort of obese youths. Previously, one study investigated zonulin levels before and after OGTT in adulthood. Saitoguillari et al. reported a positive correlation between fasting and after 2-hours zonulin in prediabetic patients versus controls (13). Moving from our results, we can argue that, during acute hyperglycemia induced by OGTT, up-regulation of zonulin is long-lasting and may affect intestinal function. In the light of the above-mentioned relationship between intestinal permeability, chronic low-grade inflammation, and obesity, increased zonulin seems to reflect not only intestinal permeability but also a reaction to inflammation and IR, establishing a persistent and self-maintaining vicious circle. Future research directions may allow advanced understanding of this relationship, which could translate clinically in the opportunity to diagnose children and adolescents at risk for metabolic disorders, even at an early stage. Furthermore, the link between intestinal permeability, inflammation and obesity enhances the rational for developing new therapeutic strategies focused on microbiome modifications (e.g., via dietary pattern modifications or probiotics administration).

We acknowledge some limitations of our study:

- the lack of a sex and age-matched control group of normal weight subjects;
- the cross-sectional design which does not allow to explore variation of zonulin levels over time, possibly after weight modifications;
- the uncertainty on the intestinal origin of zonulin, since the protein is secreted mainly from the liver and the enterocytes, but also from other tissues (adipose tissue, skin, immune cell, brain, heart, lungs, and kidney).

In conclusion, the present study provided new evidence about the relationship between serum zonulin, obesity and obesity-related complications in pediatric age, shedding new light on the emerging role of zonulin as a promising and valuable biomarker also in childhood obesity. Therefore, it is possible to suggest that zonulin might play a role not only as a marker of impaired intestinal permeability, but also as a possible indicator of early metabolic disorders in children and adolescents with overweight and obesity, helping to identify patients at increased risk of developing obesity-related complications due to IR.

Further studies, especially in pediatric age, are needed to clarify the exact role of zonulin in the pathogenesis of obesity, and to confirm whether increased zonulin levels may have negative effects on intestinal permeability.

## Data availability statement

The original contributions presented in the study are included in the article/supplementary material. Further inquiries can be directed to the corresponding author.

## Ethics statement

The studies involving humans were approved by Ethical Committee of the University Hospital AOU Policlinico “Gaetano Martino” (Protocol N.68-20, 14.10.2023). The studies were conducted in accordance with the local legislation and institutional requirements. Written informed consent for participation in this study was provided by the participants’ legal guardians/next of kin. Written informed consent was obtained from the minor(s)’ legal guardian/next of kin for the publication of any potentially identifiable images or data included in this article.

## Author contributions

GP: Conceptualization, Data curation, Formal Analysis, Investigation, Methodology, Software, Visualization, Writing – original draft, Writing – review & editing. DCo: Conceptualization, Formal Analysis, Methodology, Project administration, Resources, Supervision, Validation, Writing – review & editing. MC: Formal Analysis, Investigation, Supervision, Writing – review & editing. TA: Software, Supervision, Validation, Visualization, Writing – review & editing. AA: Formal Analysis, Writing – review & editing, Data curation, Software, Visualization. RI: Formal Analysis, Supervision, Writing – review & editing. DCa: Data curation, Methodology, Supervision, Validation, Writing – review & editing, Formal Analysis. MW: Conceptualization, Data curation, Funding acquisition, Methodology, Project administration, Resources, Supervision, Validation, Writing – review & editing.
